# Crystal and Magnetic Structures of Double Hexagonal Close-Packed Iron Deuteride

**DOI:** 10.1038/s41598-020-66669-4

**Published:** 2020-06-18

**Authors:** Hiroyuki Saitoh, Akihiko Machida, Riko Iizuka-Oku, Takanori Hattori, Asami Sano-Furukawa, Ken-ichi Funakoshi, Toyoto Sato, Shin-ichi Orimo, Katsutoshi Aoki

**Affiliations:** 1 Quantum Beam Science Research Directorate, National Institutes for Quantum and Radiological Science and Technology, 1-1-1, Kouto, Sayo-cho, Sayo-gun, Hyogo 679-5148 Japan; 2 Geochemical Research Center, Graduate School of Science, The University of Tokyo, 7-3-1 Hongo, Bunkyo-ku, Tokyo 113-0033 Japan; 3 J-PARC Center, Japan Atomic Energy Agency, Tokai, Naka, Ibaraki 319-1195 Japan; 4 Neutron Science and Technology Center, Comprehensive Research Organization for Science and Society, Shirakata, 162-1, Shirakata, Tokai, Naka, Ibaraki 319-1106 Japan; 5 Institute for Materials Research, Tohoku University, 2-1-1 Katahira, Aoba-ku, Sendai 980-8577 Japan; 6 WPI-Advanced Institute for Materials Research (AIMR), Tohoku University, 2-1-1, Katahira, Aoba-ku, Sendai 980-8577 Japan

**Keywords:** Materials science, Physics

## Abstract

Neutron powder diffraction profiles were collected for iron deuteride (FeD_*x*_) while the temperature decreased from 1023 to 300 K for a pressure range of 4–6 gigapascal (GPa). The ε′ deuteride with a double hexagonal close-packed (dhcp) structure, which coexisted with other stable or metastable deutrides at each temperature and pressure condition, formed solid solutions with a composition of FeD_0.68(1)_ at 673 K and 6.1 GPa and FeD_0.74(1)_ at 603 K and 4.8 GPa. Upon stepwise cooling to 300 K, the D-content *x* increased to a stoichiometric value of 1.0 to form monodeuteride FeD_1.0_. In the dhcp FeD_1.0_ at 300 K and 4.2 GPa, dissolved D atoms fully occupied the octahedral interstitial sites, slightly displaced from the octahedral centers in the dhcp metal lattice, and the dhcp sequence of close-packed Fe planes contained hcp-stacking faults at 12%. Magnetic moments with 2.11 ± 0.06 μ_B_/Fe-atom aligned ferromagnetically in parallel on the Fe planes.

## Introduction

Iron (Fe) reacts with hydrogen (H) to form solid solution FeH_*x*_ or stoichiometric monohydride FeH_1.0_ at hydrogen pressures (hereafter referred to simply as *pressure*) in a gigapascal (GPa) range. Because of a prototypical transition-metal hydride, structural and physical properties have been intensively investigated for the Fe–H system over the past 50 years^1–20^. In temperature–pressure (*T–P*) ranges of 0–2000 K and 0–10 GPa, three solid phases (α, ε′, and γ phases) are present: the α phase with a body-centered cubic (bcc) structure, the ε′ phase with a double hexagonal close-packed (dhcp) structure, and the γ phase with a face-centered cubic (fcc) structure^2,8,10^. These phases join at a triple point at ~570 K and ~5.0 GPa^7,10^. In each hydride, dissolved H atoms, partially or fully occupying the interstitial sites of a host metal lattice, cause the metal lattice to expand and provide a certain amount of electrons to the metal lattice^1,6,14,18^. Thus, hydrogenation is an effective means for creating or modifying the physical properties while maintaining the structure of the metal lattice.

The ε′ phase exhibits unique structural and physical properties, e.g., extensive stability, stoichiometric composition, and ferromagnetism. The phase diagram of the Fe–H system extending to 3000 K and 120 GPa indicates the ε′ phase as the only stable phase at pressures greater than 20 GPa, presumably maintaining the stoichiometric composition of FeH_1.0_^12^. Such unique phase stability allows for the investigation of the structural and magnetic properties over a wide *T–P* range. Ferromagnetic–paramagnetic transition has been experimentally investigated at ambient temperature and pressure up to 80 GPa by Mössbauer (MB) and X-ray magnetic circular dichroism (XMCD) spectroscopies^21–23^. These results showed that the magnetic moment of dhcp FeH_1.0_ continuously deceased with pressure and eventually disappeared at roughly 30 GPa at 300 K. The magnitude and alignment of the magnetic moments have been theoretically predicted at 0 K and ambient pressure using density-functional theory (DFT) calculations of the electronic band structure^24–28^.

Two essential subjects regarding the ε′ phase remain uninvestigated. First, the magnetic structure should be determined experimentally although the ferromagnetism has been confirmed by MB, XMCD, and magnetization measurements^21–23,29–34^. Neutron powder diffraction (NPD) measurements were carried out for quenched deuterides/hydrides containing the ε′ phase as a major component^6^. The crystal structure, including the H/D atomic positions, was precisely determined, but the magnetic structure was not determined because of the weak magnetic scattering intensities. Second, the H content *x* is unknown for the high-temperature solid solution. The dhcp hydride has been considered to maintain *x* = 1.0 across almost the entire stable *T–P* range^1^. The most recent X-ray diffraction measurements revealed a substantial reduction in volume at high temperatures near the dhcp*–*fcc phase boundary, and the partial release of dissolved H atoms or the formation of the solid solution was suggested in this regard^16^.

We carried out *in situ* NPD measurements on FeD_*x*_ in the *T–P* ranges of 300*–*1023 K and 4.2*–*6.1 GPa. The crystal and magnetic structures were refined for the dhcp deuteride using the model structures proposed in the early NPD study^6^ and predicted in the electronic band structure calculation^26^. The structure of dhcp FeD_1.0_ contained the off-central displacement of D atoms on octahedral interstitial sites and stacking faults in the dhcp sequence of Fe planes, consistent with the early results^6^. The atomic displacement and the stacking faults were partially or fully removed in the solid-solution states at high temperatures. The magnitude and alignment of the magnetic moments were in agreement with those theoretically calculated for ferromagnetic dhcp FeH_1.0_^26^.

## Results

**Figure 1:**
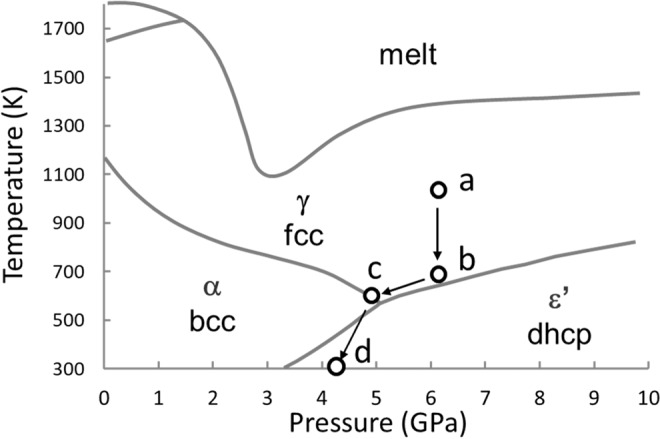
Phase diagram of the Fe–H system redrawn from^10^. A triple point is located at ~570 K and ~5.0 GPa. Open circles denote the *T–P* points of NPD measurements: (**a**) 1023 K and 6.1 GPa, (**b**) 673 K and 6.1 GPa, (**c**) 603 K and 4.8 GPa, and (**d**) 300 K and 4.2 GPa.

NPD profiles were collected for the Fe deuteride at four *T–P* points (Fig. 1). The fcc solid solution was first prepared by deuterization of fcc Fe at 1023 K and 6.1 GPa and then cooled to 673 K, where the fcc deuteride partially transformed to the dhcp deuteride along with the formation of a small amount of a metastable hcp modification. The measured *T–P* point was located immediately above the γ–ε′phase boundary and was considered to be still within the stable range of the γ phase. The latest NPD study of Fe hydride revealed that the γ–ε′phase boundary was located at temperatures 100–200 K higher than those shown in Fig. 1^19^, and the appearance of the dhcp deuteride at 673 K and 6.1 GPa was consistent with the latest result. The formation of the metastable hcp phase was sensitive to the cooling rate; a larger amount of the hcp deuteride formed at a faster cooling rate^18^. The cooling rate was gradually decreased from 10 K/min to 1 K/min. The hcp formation, however, was not completely prevented. The coexisting state of the dhcp, fcc, and hcp deuterides was retained at 603 K and 4.8 GPa. When the temperature was decreased to 300 K, the hcp deuteride decomposed to dhcp monodeuteride and bcc Fe, whereas the dhcp and fcc deuteride remained almost unchanged.

**Figure 2:**
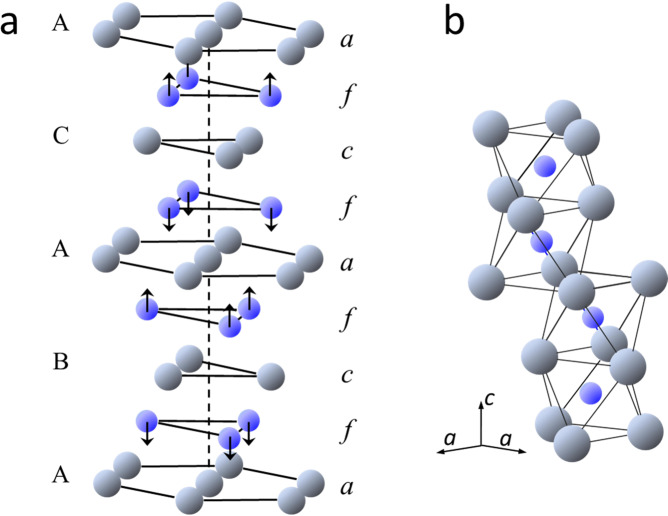
(**a**) Crystal structure of dhcp FeD_1.0_ redrawn from^6^. Here, *a*, *c*, and *f* denote the planes of equivalent positions in the P6_3_/mmc space group that originated, respectively, from the positions 2*a*, 2*c*, and 4 *f* with* z* ≈ 7/8 (see Table 1). Gray and blue spheres indicate the regular positions of Fe and D atoms, respectively. Arrows indicate the directions of displacement of D atoms from the centers of octahedral interstices. Here, A, B, and C represent the standard notation for close-packed planes. (**b**) Face-shared octahedra accommodating D atoms.

The observed diffraction profiles were simulated using model structures proposed for dhcp^6^, fcc^14^, and hcp hydrides/deuterides^18^ in early NPD studies. Dissolved D atoms were located on the centers of the octahedral and tetrahedral interstitial sites (hereafter, referred to as the O site and T site, respectively) of the fcc and hcp metal lattices, whereas they were located on the off-centered positions of the octahedra in the dhcp lattice. The crystal structure of dhcp FeD_1.0_ used as the model structure is schematically shown in Fig. 2^6^. This structure belongs to the P6_3_/mmc space group and has the stacking sequence of the Fe planes, ABACA ∙ ∙ ∙ , which comprises “hexagonal” stacking of ABA or ACA and “cubic” stacking of BAC. Figure 2b shows the spatial configuration of the Fe octahedra available for accommodating D atoms, and the octahedra are connected in a face-shared configuration in the hexagonal stacking sequence and an edge-shared configuration in the cubic stacking sequence. Two structural irregularities were considered in the refinement of the dhcp structure according to^6^, i.e., the displacement of D atoms along the *c*-axis (δ*z*) and the stacking fault of the Fe planes, which was described using *f*_reg_ and *f*_def_, denoting occupancies on the regular sites equivalent to the (1/3, 2/3, 7/8) position and defect sites equivalent to the (1/3, 2/3, 1/8) position, respectively.

For ferromagnetic dhcp FeD_1.0_, two possible structures, *in-plane* and *out-of-plane* models, were examined when refining the magnetic structure^6,26^. The magnetic moments on the Fe atoms lay parallel within the metal planes in the in-plane model, whereas they stand vertically on the metal planes in the out-of-plane model. The calculations of neutron scattering intensities for the two model structures showed that their contributions to peak intensities were limited approximately to the 100, 101, 004, and 102 peaks, and relative intensities were substantially different between the two models^6^. These calculated peak intensities served as a guideline for the refinement of the magnetic structure in this study.

**Figure 3:**
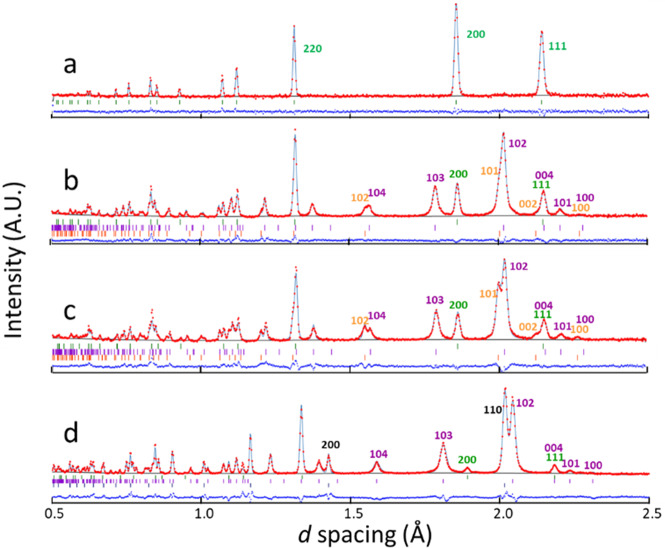
Powder neutron diffraction profiles of FeD*x* measured at the following: (**a**) 1023 K and 6.1 GPa, (**b**) 673 K and 6.1 GPa, (**c**) 603 K and 4.8 GPa, and (**d**) 300 K and 4.2 GPa. Solid lines indicate diffraction profiles simulated using Z-Rietveld^35^. Blue lines indicate differences between the experimental (dots) and simulated (curves) profiles. Reflection indices and tick marks of Bragg peaks are shown in green, purple, orange and black for fcc, dhcp and hcp FeD_*x*_ and bcc Fe, respectively.

Figure 3 shows the observed diffraction profiles and their simulated profiles fitted by Rietveld refinement using a Z-Rietveld software (Version 1.1.2)^35^. As shown in Fig. 3a, the single-phase profile at 1023 K and 6.1 GPa was fitted with the fcc structure with a composition of FeD_0.62(3)_ (hereafter, the numbers in parentheses denote experimental error). The profile at 673 K and 6.1 GPa had good a fit with the mixture of dhcp FeD_0.68(1)_, hcp FeD_0.56(2)_, and fcc FeD_0.59(1)_, with mass ratios of *x*_mass_ = 0.667(3), 0.171(2), and 0.162(2), respectively (Fig. 3b). The mass ratios slightly changed upon cooling to 603 K: *x*_mass_ = 0.564(4) (dhcp), 0.277(4) (hcp), and 0.16 (fcc) (Fig. 3c). The diffraction profile at 300 K and 4.2 GPa was fitted with the mixture of dhcp FeD_1.0_, fcc FeD_1.0_, and bcc Fe at mass ratios of *x*_mass_ = 0.708(4), 0.014(1), and 0.278(3), respectively (Fig. 3d).

Several fitting parameters were properly constrained to avoid convergence of the parameters into unphysical values. The atomic displacement parameters or temperature factors of D and Fe atoms were assumed equal among the coexisting deuterides. The occupancy ratio of *f*_def_/*f*_reg_ was constrained to be equal between the Fe and D atoms in the refinement for the dhcp structure. The D composition was fixed at *x* = 1.0 for the dhcp and fcc deuterides in the refinement of the 300 K*–*4.2 GPa profile according to the early studies^6,16,18^. In addition, the magnetic moment of bcc Fe was fixed to an ambient pressure value of the 2.1 μ_B_/Fe-atom (μ_B_ denotes the Bohr magneton). H atoms, which were included as an impurity by 4 atom% in the D source of AlD_3_, were assumed to randomly occupy the D-atom sites. For the sake of simplicity, the site occupancies of H atoms and the H composition are included in the notations *g*_D_ and *x*, respectively.

**Figure 4:**
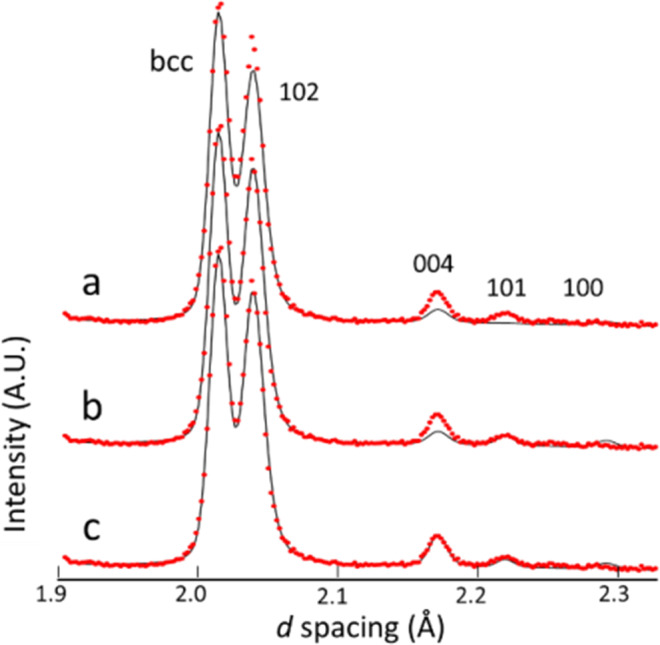
Diffraction profile at 300 K*–*4.2 GPa in the *d*-spacing range from 1.7 to 2.4 Å (red crosses). The profile was simulated (**a**) without a magnetic order, (**b**) with the out-of-plane model, and (**c**) with the in-plane model. Reflection indices denote the major peaks of dhcp deuteride.

For the dhcp FeD_1.0_ at 300 K and 4.2 GPa, crystal structure including magnetic ordering was refined using the out-of-plane and in-plane models. In both models, one equivalent magnitude of magnetic moment was assumed. Figure 4 shows the fitting results obtained with (a) the nonmagnetic, (b) out-of-plane, and (c) in-plane models. The simulated profile of the nonmagnetic model showed substantial deficits in the intensities of the 100, 101, 004, and 102 peaks. A simulated profile for the out-of-plane model reproduced, with a reliability value of *χ*^2^ = 11.3, the observed intensities of the 101 and 102 peaks but failed to reproduce that of the 004 peak. The in-plane model yielded a slightly less value of *χ*^2^ = 10.3 and satisfactorily reproduced the intensities of all peaks with an optimized magnetic moment of 2.11(6) μ_B_/Fe-atom. The rather high value of *χ*^2^ arose from misfits in peak shape likely attributed to enhanced distortion of the dhcp Fe lattice by interstitial D atoms fully occupying the O sites.

The structure refinements included preferred-orientation correction for the *c* axis of the dchp lattice. *Pref. orient*. [001] is a parameter of the March-Dollase function implemented in the z-Rietveld program^35^, and is optimized to reproduce peak intensities modified by the preferred orientation of the *c* axis. The structure refinement of dhcp FeD at 300 K and 4.2 GPa yielded *pref. orient*. [001] = 1.01. Calculations of peak intensities using a value of 1.01 and the March-Dollase function showed that the 100, 101, 004, and 102 peak intensities were modified by factors of 1.015 (+1.5%), 1.012 (+1.2%), 0.971(−2.9%), and 1.005 (+0.5%), respectively. These modification values are substantially less than the magnetic scattering components of the peak intensities as shown in Table 2 in the following paragraph: for example, the 004 peak, with a maximum modification value of −2.9%, has a magnetic scattering component 57.1%. The parameter for preferred-orientation correction dose not significantly correlate with the Bragg intensities and hence its bias to the assignment of magnetic structure can be ruled out.

**Table 1:** Structural parameters for dhcp, hcp, and fcc Fe deuterides, and bcc Fe optimized by Rietveld refinements.

*T*, *P*, Reliable factors	Phase	Atom	Site	*x*	*y*	*z*	*B*(Å^2^)	Occupancy
1023 K, 6.1 GPa *R*_wp_ = 10.2%, *χ*^2^ = 1.12	fcc-FeD_0.62(3)_, *X*_mass_ = 1.00 a = 3.70694(8)Å	Fe D D	4*a* 4*b* 8*c*	0 1/2 1/4	0 1/2 1/4	0 1/2 1/4	1.13(5) 3.3(1)	1.0 0.50(1) 0.06(1)
673 K, 6.1 GPa *R*_wp_ = 6.29%, *χ*2 = 4.42	dhcp-FeD_0.68(1)_, *X*_mass_ = 0.667(3) a = 2.63677(8)Å, c = 8.6103(5)Å pref. orient. [001] = 1.11	Fe Fe D Fe D	2*a* 2c 4 *f* 2*d* 4 *f*	0 1/3 1/3 1/3 1/3	0 2/3 2/3 2/3 2/3	0 1/4 0.8767(4) 3/4 0.1233(4)	0.74(2) 2.15(7)	1.0 0.982(3) 0.670(6) 0.018(3) 0.012(1)
	hcp-FeD_0.56(2)_, *X*_mass_ = 0.171(2) a = 2.6251(3)Å, c = 4.2478(11)Å pref. orient. [001] = 1.36	Fe D	2 *c* 2 *a*	1/3 0	2/3 0	1/4 0	0.74(2) 2.15(7)	1.0 0.56(2)
	fcc-FeD_0.59(1)_*, X*_mass_ = 0.162(2) 3.72274(11)Å	Fe D D	4*a* 4*b* 8*c*	0 1/2 1/4	0 1/2 1/4	0 1/2 1/4	0.74(2) 2.15(7)	1.0 0.59(1) 0.0
603 K, 4.8 GPa *R*_wp_ = 7.16%, *χ*^2^ = 3.38	dhcp-FeD_0.74(1)_, *X*_mass_ = 0.564(4) a = 2.63759(8)Å, c = 8.6231(7) Å pref. orient. [001] = 1.22	Fe Fe D Fe D	2*a* 2c 4 *f* 2*d* 4 *f*	0 1/3 1/3 1/3 1/3	0 2/3 2/3 2/3 2/3	0 1/4 0.8818(5) 3/40.1182(5)	0.66(3) 1.93(7)	1.0 0.957(3) 0.716(6) 0.043(3) 0.028(1)
	hcp-FeD_0.44(1)_, *X*_mass_ = 0.277(4) a = 2.61400(16)Å, c = 4.2464(6) Å pref. orient. [001] = 1.22	Fe D	2*c* 2*a*	1/3 0	2/3 0	1/4 0	0.66(3) 1.93(7)	1.0 0.44(1)
	fcc-FeD_0.46(1)_, *X*_mass_ = 0.159(2) a = 3.7227(2)Å	Fe D D	4*a* 4*b* 8*c*	0 1/2 1/4	0 1/2 1/4	0 1/2 1/4	0.66(3) 1.93(7)	1.0 0.46(1) 0.0
300 K, 4.2 GPa *R*_wp_ = 10.6%, *χ*^2^ = 10.3	dhcp-FeD_1.0_, *X*_mass_ = 0.708(4) a = 2.66727(3)Å, c = 8.7277(3) Å pref. orient. [001] =1.01 μ_mag_ = 2.11(6) μ_B_	Fe Fe D Fe D	2*a* 2c 4 *f* 2*d* 4 *f*	0 1/3 1/3 1/3 1/3	0 2/3 2/3 2/3 2/3	0 1/4 0.8803(2) 3/4 0.1197(2)	0.33(5) 0.96(2)	1.0 0.877(1) 0.877(1) 0.123(1) 0.123(1)
	fcc-FeD_1.0_, *X*_mass_ = 0.014(1) a = 3.7800(7)Å	Fe D D	4*a* 4*b* 8*c*	0 1/2 1/4	0 1/2 1/4	0 1/2 1/4	0.33(5) 0.96(2)	1.0 1.0 0.0
	bcc-Fe, *X*_mass_ = 0.278(3) a = 2.84992(4)Å μ_mag_ = 2.1μ_B_	Fe	2*a*	0	0	0	0.096(6)	1.0

Here, *B* denotes isotropic atomic displacements in the unit of Å^2^. The occupancy of Fe atoms at the 2*d* positions, which is denoted by *f*_def_ in the text, indicates the degree of stacking fault in the sequence of metal planes. The deviation of the *z*-position of D-atoms from 1/7 or 7/8 indicates off-centred displacement from the O-site centers, while* μ*_mag_ denotes magnetic moment for dhcp FeD_1.0_ and bcc Fe. Reliability values of *R*_wp_ and χ^2^ are also given. *Pref. orient.*[001] is a fitting parameter for reproducing peak intensities modified by the preferred orientation of the *c* axis.

**Table 2:** Nuclear (*I*_nuc_) and magnetic scattering (*I*_mag_) components of the 100, 101, 004, and 102 reflections of dhcp FeD_1.0_ with the in-plane model.

*h k l*	*I*_nuc_	*I*_mag_	*I*_mag_ /(*I*_nuc_ + *I*_mag_)
1 0 0	1.37	0.67	0.329
1 0 1	0.12	3.12	0.962
0 0 4	3.95	5.26	0.571
1 0 2	100	9.99	0.091

Each value isnormalized by *I*_nuc_ = 100 for the 102 peak.

The fitting parameters optimized for the dhcp deuteride and the coexisting fcc and hcp deuterides, and bcc Fe are listed in Table 1. The contributions of nuclear scattering (*I*_nuc_) and magnetic scattering (*I*_mag_) to the intensities of the 100, 101, 004, and 102 peaks of dhcp FeD_1.0_ are derived from the simulated profile shown in Fig. 4c and listed in Table 2.

## Discussion

### Crystal structure of dhcp deuteride

The structural parameters optimized for the dhcp FeD_1.0_ at 300 K and 4.2 GPa are in agreement with those reported for the dhcp deuteride quenched at 90 K and 0.1 MPa^6^. The D atoms are located at the off-center positions displaced by δ*z* ~0.005*c* from the O-site centers along the *c*-axis. The stacking sequence of the Fe planes contains the hcp-stacking faults, as described by a 0.12 occupancy of Fe atoms at the *f*_def_-defect positions. The values of δ*z* and *f*_def_ are close to 0.007*c* and 0.155, respectively, of the quenched specimen. The off-center displacement and the stacking fault are removed in the dhcp solid solutions at high temperatures. In dhcp FeD_0.68(1)_ at 673 K and 6.1 GPa, neither Fe nor D atoms occupy the defect positions, and the positions of D atoms still slightly deviate from the center of the octahedral site.

The off-center displacement is attributed to the repulsive interactions between the interstitial D atoms^6^. As known as the empirical 2.1-Å rule – dissolved H atoms in metals cannot approach each other within 2.1-Å in metal hydrides^36^, because there are strong long-range repulsive interactions between interstitial H atoms. The nearest neighboring D–D distance is calculated as 2.18 Å (close to the critical value) for the D atoms, when assumed to occupy exactly the center positions of the face-shared octahedra. The displacement of D atoms from the centers by δ*z* = 0.0053*c* (0.045 Å) increases the D–D distance to 2.27 Å, significantly greater than the critical value. As a result, some reduction in repulsive energy is expected.

The removal of atomic displacement in the solid solution is attributed to the partial release of the D atoms from the interstitial sites of the dhcp metal lattice. The D-content *x* decreased from 1.0 at 300 K to 0.68(1) at 673 K, and a third of the O sites became empty. All D atoms form nearest neighbor-pairs in FeD_1.0_, whereas roughly half of the D atoms form pairs in the solid-solution FeD_0.68(1)_ (the pairing ratio can be calculated by a square of the O-site occupancy of 0.68). The reduction in repulsive energy thus bring the D atoms back to the centers of octahedra. Another possible driving force for centering the D atoms is the thermal vibrations of the D atoms enhanced at high temperatures. The atomic displacement parameter *B* of the D atoms increased from 0.91 to 2.15 Å^2^ as the temperature increased from 300 to 673 K. This value is almost twice as large as the 1.20 Å^2^ for the H atoms in the dhcp FeH quenched at 300 K and 0.1 MPa, where no off-center displacement of H atoms was observed despite the full occupation of the O sites by H atoms^6^.

The regular dhcp stacking sequence of the Fe planes gradually recovers with elevating temperature. The occupancy of the Fe atoms at the defect sites *f*_def_, which represents the stacking fault degree, decreases from 0.123 at 300 K to 0.018 at 673 K via 0.043 at 603 K. This continuous variation with temperature contrasts the abrupt centering of the D atoms at 673 K. The atomic displacement parameter of the Fe atoms gradually increased from 0.33 to 0.74 Å^2^ as the temperature increased from 300 to 673 K. Recovery of the regular stacking sequence appears unrelated to the centering of the D atoms and would be driven by the thermal activation of Fe atomic motions.

### Magnetic structure of dhcp deuteride

The in-plane magnetic ordering determined for the dhcp FeD_1.0_ is consistent with that proposed for dhcp FeH_1.0_ in the early theoretical study^26^. The in-plane ordering of magnetic moments, proposed as a stable magnetic structure by the DFT calculations, is experimentally confirmed by the present NPD study. The calculations also predicted the magnetic moments of 1.90 and 2.08 μ_B_/Fe-atom at sites 2*a* and 2*c*, respectively^26^. In fact, two distinct values of hyperfine fields were correspondingly observed by MB spectroscopy^30–34^. In the present structure refinement, the magnetic moment of 2.11(6) μ_B_/Fe-atom is obtained under an assumption of one equivalent magnetic moment for the two sites. This value is slightly larger than the calculated moments and slightly less than the experimental moment of 2.22 μ_B_/Fe-atom for dhcp FeH_*x*_, that was measured for 0.65 ≤ *x* ≤ 0.81 at temperatures of 4.2–80 K at ambient pressure^7,29^. The magnetic moment tends to increase with both decreasing *T* and *x*^27^. The value of 2.11(6) μ_B_/Fe-atom of the dhcp FeD_1.0_ at 300 K can be interpreted in terms of these *T* and *x* dependencies.

The large magnetic moment of the dhcp FeD_1.0_ suggests a high critical temperature, Curie temperature *T*_c_, for its ferromagnetic–paramagnetic transition upon temperature elevation. High *T*_c_ is expected from the temperature-dependence of the magnetization measured for the quenched dhcp FeH_*x*_^29^. However, the measured temperature range of 4.2–80 K is too limited to estimate a *T*_c_ most likely far above room temperature. Pure iron, bcc Fe, has a magnetic moment of 2.218 μ_B_/Fe-atom at 0 K and 0.1 GPa and undergoes a magnetic transition at 1043 K. The dhcp deuteride with a comparable magnetic moment is hence expected to exhibit a corresponding *T*_c_ of around 1000 K. Unfortunately, the magnetic moment was not determined for the dhcp solid solutions at high temperatures because observation of the magnetic scattering peaks was hindered by the rather intense peaks from the coexisting fcc FeD_*x*_. The variation of *T*_c_ with *x* is essential for understanding the ferromagnetism in relation to the volume expansion and electron doping caused by dissolved D atoms. NPD investigation of dhcp deuteride, including solid solution states at high temperatures and high pressures, is a future research subject.

## Methods

Reagent-grade pure iron flakes (purity: 99.9%) with a lateral particle size <100 μm and a thickness <20 μm were used as a starting material. A compacted Fe disc 3 mm in diameter and 2.5 mm in height was prepared by pressing the flakes in a piston–cylinder-type mold. The Fe disc was placed at the center of a NaCl capsule 5.5 mm in outer diameter and 8.2 mm in height, along with the internal deuterium source of AlD_3_ (isotopic purity: 96 atom% D) pellets, placed above and below the disc. An excess amount of AlD_3_ (Fe/D molar ratio of ~1.5) was charged into the cell to form Fe deuteride with equilibrium-D compositions during NPD measurements. The NaCl capsule containing the Fe disc and AlD_3_ pellets was inserted into a cylindrical graphite heater and embedded in a pressure-transmitting medium (15-mm edge cube) made of MgO with a 5% Cr_2_O_3_ weight.

The high-pressure cell containing the Fe disc and AlD_3_ pellets was first pressurized to ~6 GPa at 300 K and then heated to 1023 K. During heating, the AlD_3_ pellets decomposed to provide a D_2_ fluid, which dissolved into the Fe specimen to form fcc FeD_*x*_. After confirming the deuteride formation at 1,023 K and 6.1 GPa by NPD diffraction, we lowered the temperature stepwise to 300 K for NPD measurements. Diffraction profiles were collected at four *T–P* points (Fig. 1), with an exposure time from 2 to 6 h, using a neutron source operating at a proton beam power of 300 kW.

The cell assembly and high-pressure apparatus used in the NPD measurements were described in detail in a previous paper^14^. Diffraction profiles were collected at the “PLANET” beamline at the Japan Proton Accelerator Research Complex (J-PARC), Tokai, Japan^37,38^.

## Data Availability

All data supporting the findings of this study are available within the paper and Methods. The crystallographic data are available from the corresponding authors upon request.
